# Supplementary material for: Learning curve and surgical outcome of robotic assisted colorectal surgery with ERAS program
Source: Sci Rep. 2022 Nov 29;12:20566. doi: 10.1038/s41598-022-24665-w (PMC9709162; doi:10.1038/s41598-022-24665-w)
Supplement: Supplementary file 1 — Supplementary Information 1. [file 41598_2022_24665_MOESM1_ESM.zip › LSC Raw data-Ñ[▒K/132/O_ñw╝╨░O▒Kñσ.pdf]

正式摘要(電子簽章：000000223371404)

請選擇...reporter

臺中榮民總醫院

Operation Note

[redacted]

- 手術日期：2021/08/11
- 手術醫師：VS 林俊余，CR 陳映菁，R 郭連雄
- Anesthesia：GE
- Position：Lithotomy
- Aseptic agents：Soap，Betadine Alcohol，Betadine Aqua

Diagnosis

- Pre-operative diagnosis : CA OF S-COLON FOR LAPA-OP
- Post-operative diagnosis : Sigmoid colon cancer with small intestine mesentary invasion, s/p laparoscopic converted to conventional Anterior resection on 20210811,cT4N1M0

Surgical Indication

- For curative resection

Operation

- 1. Laparoscopic converted to conventional anterior resection due to advanced tumor invasion to mesentary of small intestine, with side-to-end anastomosis, endo-GIA 60/3.8mm*1, EEA 29mm, re-inforcement with silk: nil, bilateral doughnuts: intact, air leak test: done, no air leak.
  2. High ligation (+): IMA above left colic artery.
  3. Reperitonealization: nil.
  4. Take down of splenic flexure(+).
  5. One 12Fr. Penrose drain at pelvis.

Finding

- 1. S-colon tumor in vade to mesentary of small intestine, T4N1M0
  LAR clamp, endo GIA 60-4.1mm*2 for distal rectum
  endo GIA 60-3.8mm*1 for S-colon
  2. There was no visible liver metastasis, and no visible peritoneal seeding.
  4. thermal injury to left ureter, consulted GU doctor for D-J insertion

Procedure

- General endotracheal anesthesia was induced and then the patient was placed in modified Trendelenburg lithotomy position. Preoperative antibiotics were given. A Foley catheter and nasogastric tube were placed. The abdomen and perineum was prepped and draped in standard sterile fashion. A small vertical incision was made above the umbilicus and a 12mm trocar was inserted into the peritoneal cavity by open method. Peumoperitoneum was established with CO2 inflation. Laparoscopy was inserted and then a 12mm trocar was inserted over RLQ of abdomen. Three 5mm trocars were inserted over RUQ, LUQ and LLQ of abdomen. The liver, omentum and peritoneum were inspected for evidence of metastatic disease. No metastatic disease was noted. A mass lesion was noted in the sigmoid colon which invaded to mesentary of small intestine. Due to locally advanced tumor, we decided to convert the operation to conventional method. Paraumbilical incision was extended as lower midline incision. The small bowel was wrapped with moist pad and placed at upper abdomen, peritoneum of mesentary of small intestine was scored with electrocautery. The sigmoid colon was then held by grasper and the peritoneum overlying the mesentery was then scored with electrocautery. The inferior mesentery artery was identified, ligated above the left colic artery btween ligature and then was transected, so was inferior mesentery vein.

- Dissection was done above the Gerota’s fascia and toward the peritoneal attachments. The arcade vessels to the sigmoid colon were also divided. Dissection was done above the Gerota’s fascia and toward the splenic flexure. The colon was then freed from its peritoneal attachments along the line of Toldt proximally from the splenic flexure and distally to the pelvic inlet. Using harmonic, omentum was freed from the transverse colon. Lesser sac was opened, and splenocolic ligament was dissected, splenic flexure was taken down. A linear stapler was then used to divide the rectum at least 5 cm distal to the palpable lesion. LAR clamp was used to clamp rectum, endo GIA 60-4.3mm*2 were used to transect rectum. The distal D-colon was transected with endo GIA 60-3.8mm*1. The specimen was removed. The proximal colon was carefully inspected, noted to reach easily into the pelvis, and to have an excellent blood supply. It was determined that a 29 mm circular stapler could be accommodated. The anvil was inserted to distal D-colon, A pursestring suture of 3-0 Prolene was placed at D-colon, and then the pursestring suture was tied. The anal canal was irrigated with distil water and then the circular stapler was introduced through the anus and pierced through the rectal stump. The proximal bowel with anvil in place was engaged with the circular stapler and the stapler was then fired. Two complete donuts were obtained and submitted to pathology. The anastomosis was tested for leaks with irrigation solution and air introduced through the rectum and was found to be patent and intact. Penrose drain was placed into pelvis. The fascia of midline wound was closed with interrupted sutures of No.1 Vicryl. The fascia of the 12 mm trocar was closed with 1-0 Dexon. the skin was closedd with 3-O nylon. The patient tolerated the procedure well and was brought to the POR in stable condition.
- Estimated blood loss: minimum
- 器械數量: 正確
- 紗布數量: 正確

Image

- PAINT
  - colon & rectum

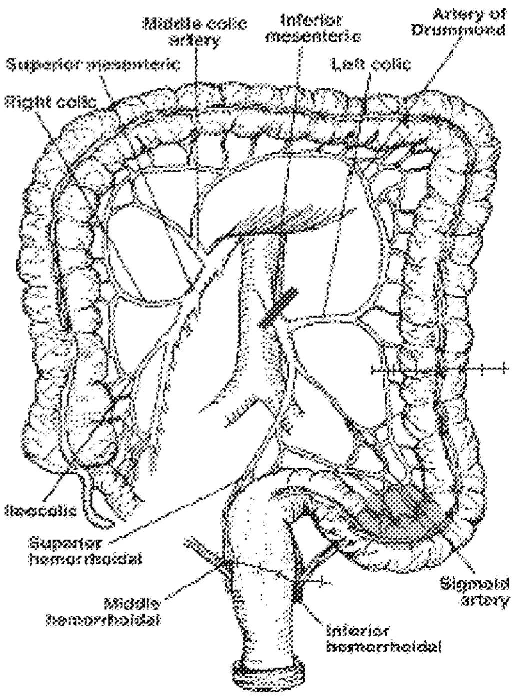

  - abdomen

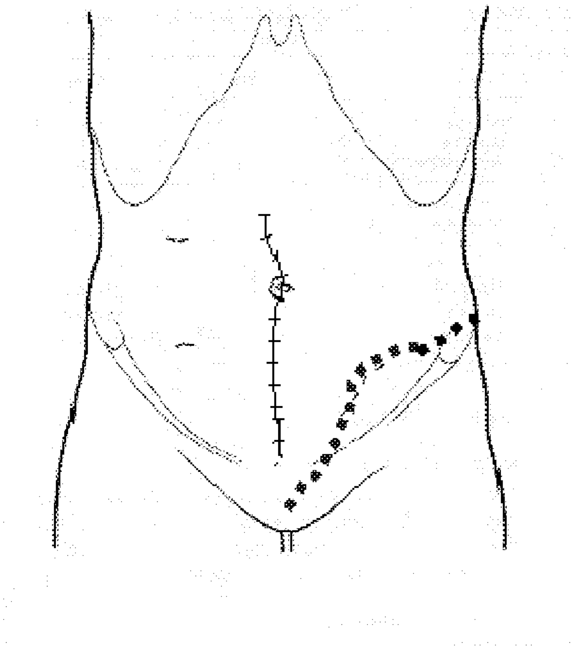

主治 2222H林俊余 / 醫師 2303K陳映菁

正式摘要(**電子簽章：000000223371404)**

請選擇...reporter

doctorRank:B
